# Metal Hypersensitivity in Joint Arthroplasty

**DOI:** 10.5435/JAAOSGlobal-D-20-00200

**Published:** 2021-03-12

**Authors:** Johannes Michiel van der Merwe

**Affiliations:** From the Adult Reconstruction Orthopaedic Department, University of Saskatchewan, Saskatoon, Saskatchewan, Canada.

## Abstract

Metal hypersensitivity in joint arthroplasty is a very controversial topic with limited evidence. With increasing numbers of joint replacements being done annually, a clear understanding of the pathogenesis, clinical picture, preimplant screening, postimplant workup, and treatment plan is crucial. This review article looked at all the available evidence regarding metal hypersensitivity and summarized the key findings. An algorithm was also proposed for preimplant screening, postimplant workup, and management.

Metal hypersensitivity (MH) is one of the most contentious and difficult topics in total hip and knee arthroplasties.^1^ Failure due to MH may not only have medical but also have legal implications.^2^ MH is especially more looked at in total knee arthroplasty. One of the reasons is that almost 20% of patients with a well-fixed, properly aligned knee implant remains dissatisfied with the procedure.^3^ Cutaneous MH in the general population is estimated to be between 10% and 15%, although it is considered to be as high as 25% in patients with metallic implants.^1,4-6^ The actual incidence of MH to metal implants is less than 1%.^7^ In a meta-analysis done in 2012, it was demonstrated that patients have a higher probability of developing a metal allergy after a failed total hip arthroplasty (odds ratio, 2.76; 95% confidence interval, 1.14 to 6.70) compared with well-functioning prostheses.^6,8^ Other studies also confirmed that MH was present in 25% of patients with a well-functioning joint arthroplasty compared with 36.9% in those with failed implants.^3,5,9^ The frequency of MH was higher in patients with failed prostheses or metal-on-metal articulations.^6^ One study mentioned that the prevalence of hypersensitivity (HS) depended on the presence and status of a joint replacement (higher rates of MH postoperative and in failed implants), the type of coupling (higher rates in metal on metal couplings), and the amount of haptens tested.^6^ In addition, patients with cutaneous MH do have a higher failure rate (up to 60%) of their total joint arthroplasty.^1^ The crucial question, however, remains whether the poorly functioning implants failed because of MH, or perhaps, the patients became sensitized because the implants failed.

## Potential Causes

A high prevalence of contact allergies to metals exists with up to 24.4% to nickel, 8.8% to cobalt, 5.9% to chromium, and 0.2% to 3% to Titanium.^10,11^ It is thought that jewelry is the most common cause of exposure, but patients can be exposed to metal in various ways.^12^ This can occur through the skin in the form of cell-phones, clothing fasteners, and leather.

It can also occur through occupational exposure such as concrete, leather work and tanning, janitorial, hairdressing, textile industry, agriculture, mechanics, metal work, dental fillings, and medical implants. Finally, it can occur through trace metal exposure in smoking, cosmetics, food, and drinking water.^5^

## Elements Used in Total Hip and Knee Arthroplasties

The most common orthopaedic implants consist of 316L stainless steel (19% chromium and 14% nickel), cobalt-chromium-molybdenum (67% chromium, 30% cobalt, 2% molybdenum, and 1% nickel), and titanium alloys (91% titanium, 5% aluminum, 3.9% vanadium, and 0.1% nickel).^10^ Nickel, being the metal with the highest percentage of metal sensitivity, is usually included in orthopaedic implants because it grants necessary strength and durability to implants.^13^ The different metal ions released during corrosion can lead to different reactions, that is, nickel can lead to implant loosening, whereas chromium is more associated with skin problems.^10^ Polyethylene and polymethyl-methacrylate particles are relatively large and do not cause the same immune response as metal ions.^3,14^ Polyethylene seems to not cause contact dermatitis, and no data exist suggesting the existence of polymer allergy.^2^

Sensitization to bone cement, however, has been reported. The cement components most frequently causing sensitivity included benzoyl peroxide (initiator) and N,N-dimethyl-p-toluidine (activator).^9^

## Pathogenesis

**Figure 1:**
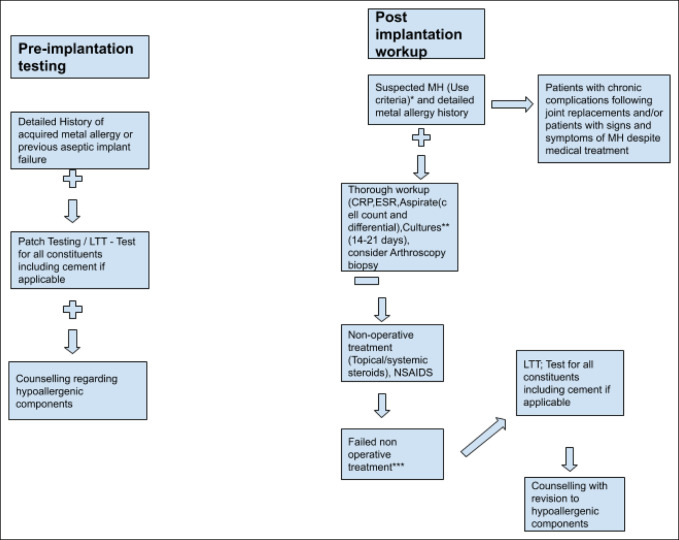
Figure demonstrating the *major criteria: cutaneous eruption overlying a metal implant, chronic dermatitis that occurs within months after the surgery, complete recovery after removal of the metal implant, and a positive patch testing to a metal used in the implant. Minor criteria: treatment resistant allergic dermatitis, positive leucocyte transformation test, morphology and histology consistent with allergic contact dermatitis, and unexplained pain at the implant site and/or failure of the implant. **The second culture should only be done if the first culture was negative. ***Persistent dermatitis with joint synovitis and/or component loosening. CRP = C-reactive protein, ESR = erythrocyte sedimentation rate, LTT = lymphocyte transformation test, MH = metal hypersensitivity, NSAID = nonsteroidal antiinflammatory drug.

MH is a type IV HS reaction (Figure 1). The difference between a type IV HS reaction and a type I or II HS reaction is that no or very small amounts of wear particles or inflammatory infiltrates are seen histologically in type IV reactions.^15,21^ Periodically, slight lymphoplasmacytic infiltrates may be detectable in type IV HS reactions in the neosynovium. The reason for this might be multifactorial (implant instability, poor alignment etc.].^15^ Type I HS reactions demonstrate macrophages and multinucleated giant cells, in which particle wear can be detected. Particles greater than 5 μm are more likely to be found in multinuclear giant cells, whereas smaller particles are seen in macrophages (<1 μm). Type I HS reactions occur when an antigen cross-linked with immunoglobulin E causes mast cell degranulation with release of vasoactive biomolecules that causes a response in seconds to minutes (anaphylaxis).^2^ Type II HS can be diagnosed histologically by the detection and quantification of neutrophil granulocytes per high power field.^15^ This can either be done via frozen sections or analyzing paraffin-embedded sections. Type III HS is a combination of type I and II HS, demonstrating histological findings of particle wear and infectious infiltrates.

Type IV MH, metal components implanted undergo some degree of galvanic corrosion, when dynamic or static metal components are in contact with liquids contained in tissues, with subsequent metal ions released. Cadosch et al reported evidence of growth and differentiation of osteoclast precursor cells on the surface of stainless steel, titanium, and aluminum implants. The mature osteoclasts then corrode the metal surfaces, which leads to metal ion release.^5,16,17^ Corrosion can also be caused by multiple other mechanisms, that is, crevice and fretting processes. Dynamic components have additional wear debris that can predominantly localize in the periprosthetic tissues. The persistency of the antigen in the periprosthetic tissues may activate a localized immune response, which could lead to implant failure.^6^ Even more important than the rare skin manifestations secondary to total joint replacements are the effects on the implant bone interface. The inflammatory response is proportional to the particulate load; metal particles are more proinflammatory and wear particle generation can cause osteoclastic activation via macrophage ingestion.^2^ This can lead to implant loosening. Metal ions bind with proteins which are identified by antigen-presenting cells. The antigen-presenting cells show the metal-antigen complex to the T-cells, which leads to a subsequent activation of CD4+ and CD8+ and macrophage cells with ensuing release of proinflammatory cells.^18^ Proinflammatory cells lead to an immune response which could cause soft-tissue inflammation with subsequent periprosthetic tissue damage.^1,3^ The osteolytic lesions found surrounding the orthopaedic implants are postulated by many studies to involve Titanium ion–induced expression (resulting in osteoclast precursor recruitment to the periprosthetic region) and cytokines released by the proinflammatory cells (promoting osteoclast differentiation and activation).^5,19,20^ The cells responsible in the periprosthetic joint, causing these metal-allergen complexes are not In tknown, which is in contrast with the skin HS reactions, where we have identified Langerhans cells in the dermis as the cause.^3^ Local tissue samples taken at the time of revision demonstrated perivascular lymphocyte infiltration, high endothelial venules, and tissue necrosis, which is a similar finding in failed metal on metal hip replacements.^2,21^ Controversy exists whether aseptic lymphocytic vasculitis (ALVAL) seen in metal-on-metal (MoM) articulations is a pathological feature of MH. Willert et al^22^ described the term ALVAL as an intense perivascular lymphocytic infiltrate that occurs around certain MoM articulations. Its histological appearance is similar to a delayed HS response. It is mainly characterized by areas of coagulated necrosis, subsurface band-like infiltrates of macrophages and giant cell granulomas, and perivascular aggregates of lymphocytes (predominantly B-cell). By contrast, MH is a T-cell–mediated inflammatory response. This cell-mediated inflammatory response can persist in the presence of a continuing source of metal ions. These metal ions (haptens) bind to proteins to form hapten-protein complexes, which lead to a cell-mediated inflammatory response. In vitro testing does not always correlate with ALVAL lesions, with minimal reactivity to cobalt and chromium in MoM articulations, despite high serum metal ion levels.^6^

## Clinical and Radiographic Assessments

MH most commonly affects women 2 months to 2 years after a joint replacement.^5,23,27^ Consistently, it occurs within the first several months after implantation.^9^ Women tend to have a higher rate of metal sensitization to most of the metals compared with men. Chromium is the only metal in which men tend to have a higher rate of MH.^4^ This is thought to be a consequence of traditional occupational exposure. Women are estimated to have a 17% incidence of MHS to nickel compared with 3% in men. This is in stark contrast to cobalt and chromium, where the incidence in the general population is much lower (1% to 2%).^24,25^ Thyssen et al^25^ concluded that the prevalence of MH is independent from age, race, or geographic location. Chromium HS is mostly associated with the concrete industry, leather work, tanning, and janitorial industry.^4^ With increasing age, a decreased risk exists of developing MH to nickel.^5,26-28^ Patients can present with swelling, limited range of motion, and pain in the affected joint. The two most commonly cited symptoms include pain and swelling.^9^ Patients also present with a localized or generalized rash, which can be present in up to 35% of patients who develop MH.^9^ Cutaneous rashes are more common in total knee arthroplasties compared with total hip arthroplasties. The rash can be papular, erythematous, pruritic, or scaly. It usually presents lateral to the midline skin incision.^3^ It can initially be localized to the knee (in a total knee arthroplasty) but then become generalized over the whole body. Patients can also present with generalized arthralgias. Radiographically, periprosthetic lucent lines can eventually occur with resultant aseptic loosening.^5^

## Testing Modalities Available

Preimplantation and postimplantation testing can be through skin patch testing, lymphocyte transformation testing (evaluation of [3H]-thymidine uptake in lymphocytes after contact with specific allergens), modified lymphocyte stimulation testing (assesses the expression of specific receptors on circulating mononuclear cells after stimulation with metals), leucocyte migration inhibition testing (measures the speed of migration of leucocytes after being in contact with sensitizing allergens), confocal microscopy (evaluates intracellular abnormalities after contact with metals), and Memory ELISA (measurement of cytokines released by stimulated cells).^1,7,29^ The two most commonly used methods used for testing include: skin patch testing (in vivo) and lymphocyte transformation test (in vitro testing).^9^ All these tests do have limitations, and none is considered to be the benchmark. Skin patch testing is, however, simple with a low cost and an excellent initial test for patients with a metal sensitivity and has a low risk to the patient.^5,12^ Potential disadvantages include different mechanisms of reactivity than that seen in deep tissues, the cost and time associated with the test, the difficulty to report quantifiable and standardized results, and the process of in vivo patch testing that could induce sensitization in a previously nonsensitized patient, leading to failure due to MH.^5,9^ In contrast to skin patch testing, lymphocyte transformation test is more sensitive and cannot induce HS, with testing seeing that it is done in vitro.^5^ Lymphocyte transformation test, however, is not readily available, and only a few allergens are tested which limits evaluation.^5^ However, controversy exists about the validity of skin patch testing to determine deep tissue or joint HS to metals.^3^ According to previous reports, skin patch testing has a sensitivity as high as 100% but a specificity as low as 64%.^10^ Skin patch testing is usually done by dermatologists who use a panel of cutaneous patch testing to different metal-salt complexes. The patches are usually removed 48 hours after placement. The erythematous reactions to the allergen are then rated at 48, 72, and 96 hours or more after placement.^3,12^ The reactions are categorized as 1+, 2+, or 3+. Patch test reactions rated 2+ or 3+ are more likely to cause implant reactions than 1+ patch testing.^11^

## Preimplantation Screening

**Figure 2:**
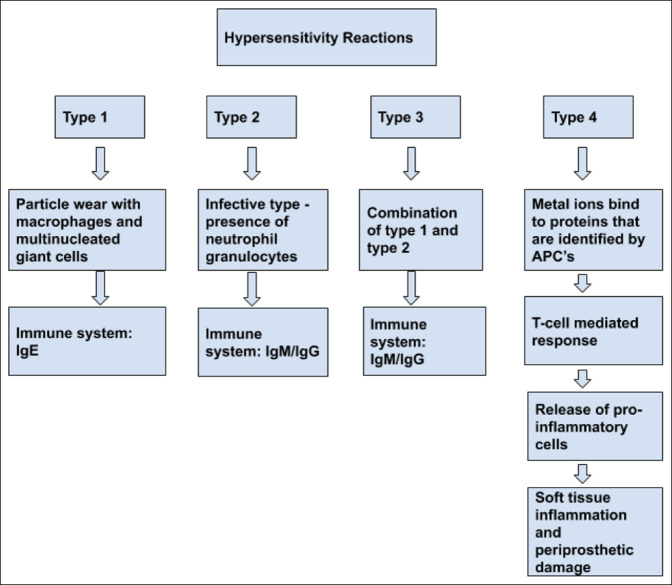
Figure demonstrating different types of hypersensitivity reactions. APC = antigen-presenting cell, Ig = immunoglobulin.

It is very controversial whether screening should be done preimplantation because of lack of evidence^18^ (Figure 2). In a 2016 cohort study, patients who tested positive for patch testing preoperatively had a similar rate of revision compared with patients testing negative.^12^ Similarly, patient screening questionnaires preoperatively have been found to have a 60% positive predictive value.^12^ The American Contact Dermatitis Society released a statement in 2016 stating that routine preoperative testing of patients without a previous history of metal allergy is unnecessary.^18^ No link also exists between development of cutaneous manifestations after joint replacement who had a positive preoperative metal sensitivity.^2^

Therefore, it is important to only do preoperative skin patch testing in patients with a notable history of acquired MH or sufficient concern based on a review of the systems. It is important, once the surgeon decides to embark on preimplantation testing, to test for all the constituents of the proposed implant and for cement where applicable. If patch testing is positive, patients should be counseled appropriately preoperatively about alternatives to standardized components and the limited evidence suggesting good outcomes with using hypoallergenic components.^9,12^ Currently, the consensus is that preoperative screening should only be done in patients who have a notable history of metal allergy.^8,12,18^ It is important to know that preoperative screening does not necessarily mean that patients will get MH after a joint replacement, and no data exist in predicting success in joint replacement using hypoallergenic components in patients testing positive for MH.^1,12,18^ On the contrary, one study looked at 21 patients with a positive patch test to metals and underwent surgery using hypoallergenic components and demonstrated no morbidity due to HS.^30^

Preimplantation screening should only be done in patients with a history of acquired MH or sufficient concerns based on a review of the systems. Skin patch testing is reasonable preimplantation because of its low cost and availability, but limited evidence regarding success in using hypoallergenic components in patients should be considered by surgeons and patients alike.

## Postimplantation Workup

Patients should be worked up properly because of the large differential diagnosis (infection, loosening, instability, and recurrent hemarthrosis) for a painful joint arthroplasty with swelling and limited range of motion (Figure 2). Criteria exist proposed by an international group of physicians to help diagnose MH postimplantation. Major criteria include cutaneous eruption overlying a metal implant, chronic dermatitis that occurs within months after the surgery, complete recovery after removal of the metal implant, and a positive patch testing to a metal used in the implant. Minor criteria include treatment-resistant allergic dermatitis, positive leucocyte transformation test, morphology and histology consistent with allergic contact dermatitis, and unexplained pain at the implant site and/or failure of the implant.^12^

The workup should include a detailed allergy-specific history, C-reactive protein and erythrocyte sedimentation rates, and joint aspiration for cell count and differential. Two aspirates done at different times for anaerobic and aerobic cultures are preferably cultured for 14 to 21 days. The second aspirate should only be done if the first culture was negative. Metal ions do not routinely have to be done seeing that they can be elevated in well-functioning total knee replacements.^3^ Some suggest doing an arthroscopic inspection to obtain biopsies for microbiology and histopathology.^23^ The arthroscopic biopsy of the neosynovium (new layer around the joint space) can be a reflection of the periprosthetic membrane (thin connective tissue layer between the bone and the implant) and therefore might play a crucial role in prerevision diagnostics. The only other way to obtain a sample of the periprosthetic membrane is to remove the components.^15^

Patch testing postimplantation as part of the routine workup is certainly more controversial. No difference exist in the long-term outcomes in patients whether nickel-containing components were used with a positive patch test to nickel.^12,31,32,38^ Other studies demonstrated shorter lifespans of implants or a higher prevalence of allergy to metals in patients undergoing two or more revisions.^12,33,34^ Higher rates of patch testing were also established in patients with dermatological reactions (47% to 67%) postimplantation and in patients with osteolysis on radiographs.^9^ However, studies exist that did not see any difference in the prevalence of positive patch test results in patients with and without joint arthroplasties.^22,35^ Other studies did not detect any increased periprosthetic loosening in patients with a positive patch test post implantation.^22,35^ Lymphocyte transformation testing demonstrated higher positive results in patients undergoing revisions or radiographic findings of osteolysis.^9^ Theories as to why the proportion of positive tests increase after joint replacement include that continued contact with metal ions released from the implant may elicit a HS reaction. Evidence did demonstrate that metal ions do increase after joint replacements, with even higher numbers of ions after failed prostheses.

Postimplantation testing for MH is recommended for patients with chronic complications after arthroplasty or signs and symptoms of MH that persists despite medical therapy.^12^ It is crucial to test for all the metals involved in the implanted prostheses and cement in cemented components.^21^ An important issue to remember in postimplantation patch testing is prosthesis-induced sensitization.^13,36^ The prevalence of contact skin sensitivity in patients with a joint replacement device is higher than that in the general population.

Therefore, an objective determination of metal sensitivity at the preimplantation assessment should be considered when planning an arthroplasty procedure.

MH is a diagnosis of exclusion. Postimplantation testing should only be considered in patients with chronic complications where other causes have been excluded or symptomatic treatment of MH failed. Leucocyte transformation testing should be considered because of better sensitivity in comparison to skin patch testing.

## Treatment of Patients With Metal Hypersensitivity

In treating patients with MH, all other causes should be excluded and MH should be a diagnosis of exclusion.

Choosing implants in patients with proven MH undergoing a primary joint replacement is very controversial. Some surgeons use hypoallergenic components, whereas others advocate to use standard implants because of the lack of evidence supporting hypoallergenic components (only short to midterm results are available) and the difficulty in accurately diagnosing metal allergies. Currently, no definitive evidence is available to help the surgeon in decision-making. If a patient undergoes a total knee arthroplasty and develops symptoms, then symptomatic treatment (topical steroids for rashes and nonsteroid anti-inflammatories for joint pain and swelling) can have some success. Patients with persistent dermatitis (localized/systemic) should be referred to a dermatologist for treatment with topical or systemic steroids. Limited data are available recommending revision for severe systemic dermatitis. If associated joint synovitis or component loosening exists, the mainstay of treatment will be revision surgery to hypoallergenic components.^3^ Surgery should be undertaken with caution, and patients should be counseled before surgery regarding unpredictable outcomes. Revising components on the basis of pain alone without a cause of failure is unlikely to resolve the symptoms and unlikely to benefit the patient.^2^ If the surgeon embarks on surgical treatment for MH, the surgeon should revise all the components involved in the MH to hypoallergenic components and/or noncemented components.^7,29^

Hypoallergenic components are made of inert materials without immunogenic activity. Coated components use standard cobalt-chromium components coated by one or more layers of immunogenic-inert substances.^29^ Examples include the usage of ceramic on polyethylene, ceramic on ceramic-bearing surfaces in total hip arthroplasty, and an all polyethylene tibial component—Ti alloy or zirconium nitride (Aesculap) tibial component and a Ti nitride (Corin), oxidized zirconium (Smith and Nephew), or zirconium nitride femoral implant in total knee arthroplasty. Interestingly, a recent randomized controlled trial comparing coated and standard total knee arthroplasty components demonstrated only an increase of chromium at the one-year follow-up. No difference exists in serological levels of cobalt, molybdenum, and nickel. The chromium levels were statistically notable but not clinically relevant, although one study demonstrated an association between chromium and the development of excema.^3,4^

Even by revising the components, nickel can still be found in the periprosthetic tissues. This can be caused by the stainless-steel instruments containing nickel (nickel content ranges from 10% to 14%) being used during the revision surgery.^3^ Traces of nickel are also present in titanium alloys. The significance of these traces of nickel from the titanium alloys and stainless-steel instruments remains unknown.^9^

First-line treatment should include symptomatic treatment. The surgeon should only embark on surgical treatment if a clear cause exists. Patients should be appropriately counseled regarding limited evidence available in MH and the use of hypoallergenic components. If surgery is undertaken for MH, then use hypoallergenic components for metal allergy or noncemented components for cement allergy.

## Summary

MH is a controversial topic with limited evidence. It is more commonly observed in failed arthroplasties. The most common metal involved in MH is nickel. Orthopaedic implants contain nickel because of its ability to provide increased strength and durability. MH is a type IV HS reaction. Women are mostly affected and can present with pain and swelling in the early months after undergoing a joint replacement. Rashes occur in 35% of patients and are more commonly seen with knee replacements. Currently, preoperative screening should be limited to patients with a history of metal sensitivity. A thorough workup should be done postimplantation to rule out the large differential diagnoses for a failed/failing implant. MH is a diagnosis of exclusion. No gold standards exist for testing for MH, but the two most commonly used tests are patch testing and lymphocyte transformation tests. Once a diagnosis of MH is made, then treatment should be tailored to nonoperative means mainly, but if the surgeon embarks on surgical treatment, it is crucial to counsel patients appropriately regarding the limited evidence associated with hypoallergenic components to obtain more realistic expectations. Hypoallergenic components should be used in the revision setting ensuring all the metal constituents that demonstrated MH is eliminated. A lot of unknown factors with MH exist, and more research is needed to gain a better understanding of managing this challenging problem.

**Take-Home Messages**

- MH is a type IV HS reaction.
- Preoperative screening should only be limited to patients with a history of metal sensitivity.
- A thorough workup should be done in failing/failed implants suspected of MH to exclude the large differential diagnosis.
- MH is a diagnosis of exclusion.
- Hypoallergenic components have very limited evidence in treatment for MH.
